# Treatment of Erysipelas, with Iron

**Published:** 1853-10

**Authors:** G. W. Balfour


### ECLECTIC DEPARTMENT.

### *On the Treatment of Erysipelas with Iron.*

BY G. W. BALFOUR, M. D.

In consequence of having witnessed the cure of a case of erysipelas treated with iron, under the joint care of Dr. Charles Bell and myself, and also in consequence of Dr. Bell's assurance of its great and unfailing success, I was induced to give this method a trial, and this the more readily that opinions were very much divided regarding the proper treatment of this disease, and I myself had no great confidence in any. Since that time I have treated all my cases, upwards of twenty, with iron, and have had no cause to regret my doing so. On the contrary, erysipelas is one of the few diseases for which I now believe we have a certain and unfailing remedy, and this whether it be infantile or adult, idiopathic or traumatic. The first case so treated was a highly scrofulous woman, with erysipelas of the scalp, arising from irritation of two large sores on it. She was cured in three days. The second was a man with erysipelas of the foot and ankle; cured in two days. The third was a case of traumatic erysipelas of the scalp in a woman; she was ill for a day or two before being seen, but was cured in about five days' treatment. The wound, a deep cut about three inches long, was healed within a week. In short, all the cases, many of them very severe, and accompanied with high delirium, some of them phlegmonous, others vesicular, and several occurring in children, were cured in less than a week on the average. Suppuration took place in none but two—in both of whom the treatment was not commenced till after effusion had taken place. Their convalescence was, of course, more tedious. The ninth day was that on which convalescence commenced, even in the most severe cases, and *probably* the course of the disease would have been shorter even in them had the medicine been given more regularly; for so sure is it in its effects, that I can, with I may almost say absolute certainty, predict the state of the patient on ascertaining the quantity of the drug taken, and a glance at the bottle is fully as informing as to the state of the disease as a look at the affected parts themselves.

The tincture of the muriate is that preparation of iron I have hitherto employed, the dose varying with the age of the patient; the great object being to saturate the system with iron as speedily as possible, and to keep it so till the disease is abated. A few doses suffice to remove the pain, and lessen the heart's action; it acts also as a diuretic, and to some extent corrects the secretions, often cleaning the tongue as well as any purgative. It never produced headache nor other unpleasant symptom, and was continued with advantage throughout the highest delirium. The only other remedy employed was an occasional purgative, and the local application sometimes of a warm poultice, and at others of simple flour or starch powder and cotton wadding, the poultice being preferred, perhaps without much reason, when the situation or extent of the part affected did not throw difficulties in the way of its application.

Its principal curative action seems, as Mr. G. Hamilton Bell supposes, to be exerted on the capillary vessels; and for this reason, that while its diuretic action is equally well maintained by smaller doses, less frequently repeated, its curative action is not obtained unless the system be saturated with it, and kept so for some time. Further, without denying that it may act as a renal purgative in diseases, I may add that, from some experiments on myself, it does not seem to do so in health. The quantity of the urine was much increased by fully a half, yet the quantity of urea was increased relatively and absolutely only to so small an amount, a grain or two in twenty-four hours, as to be far within the usual limits of aberration, and much too insignificant, supposing it to be real, to account for the wonderful curative agency of the drug. On taking twenty minims of the tincture every two hours, the second and third doses produced some slight degree of tension in the head, a symptom which subsequently ceased, and did not return. At this period, too, the pulse was slightly accelerated, possibly from nervousness, it afterwards came down from 80, my usual average, to 64. The iron was taken at intervals of four days, regularly every two hours for the last twelve hours. Its diuretic action was always established within an hour after taking each dose; no iron could be detected in the urine, nor in the serum of the blood; but as this may depend on my want of refinement in chemistry, I am willing to give any chemist who may be anxious, another opportunity by repeating the experiment; for I know no method of treating any disease more worthy of investigation, and none more deserving of adoption by the profession than the treatment of erysipelas with iron. Some have supposed its efficacy to be owing to its *stimulant* action.—That this is not the case was well shown in a case I had recently under my care,—a young man laboring under scarlatina, who had a patch of erysipelas vesicular over the sacrum, and one over the right armpit. Neither of these were at all influenced by the carbonate of ammonia, employed in the treatment of the coexisting scarlatina, but yielded at once to twenty-four hours' treatment with the muriate of iron.—*Monthly Journal.*

